# Heat Treatment as a Safe-Handling Procedure for Rift Valley Fever Virus

**DOI:** 10.3390/pathogens13121089

**Published:** 2024-12-10

**Authors:** Maria Anele Romeo, Eliana Specchiarello, Cosmina Mija, Verdiana Zulian, Massimo Francalancia, Fabrizio Maggi, Anna Rosa Garbuglia, Daniele Lapa

**Affiliations:** 1 Laboratory of Virology, National Institute for Infectious Disease “Lazzaro Spallanzani”—IRCCS, 00149 Rome, Italy; 2 Microbiology and Virology Unit, San Gallicano Dermatological Institute, IRCCS, Istituti Fisioterapici Ospedalieri (IFO), 00144 Rome, Italy

**Keywords:** Rift Valley Fever virus, RVFV, heat inactivation, temperature, safe-handling procedures, virus stability

## Abstract

Rift Valley Fever virus (RVFV) is a mosquito-borne virus with high pathogenic potential in ruminants and humans. Due to its high potential for spreading, it is considered a priority pathogen, and it is included in the Bluepoint list of the World Health Organization (WHO). Given the high pathogenic potential of the virus, it is crucial to develop a rapid heat-mediated inactivation protocol to create a safer working environment, particularly in medical facilities that lack a biosafety level 3 laboratory required for direct handling of RVFV. Our results reveal the broad tissue tropism of RVFV, showing the virus’s capacity for replication in various cell lines. In terms of the thermal stability of RVFV, our findings showed that a 70 °C heat treatment did not fully inactivate the virus within 15 min. However, when exposed to 80 °C and 95 °C, the virus was completely inactivated after 15 min and 5 min, respectively. Additionally, our results indicated that heat-treatment only slightly decreased the integrity of the RVFV genome whether there is a high or low number of viral RNA copies. Overall, the study established a straightforward protocol for heat inactivation that may be beneficial in handling clinical and research samples of RVFV.

## 1. Introduction

The Rift Valley Fever virus (RVFV) is transmitted by mosquitoes and is a member of the Phlebovirus genus, within the Phenuiviridae family and Bunyavirales order. First identified in 1931 amidst an epidemic in livestock in Kenya’s Rift Valley, RVFV was associated with a significant increase in abortion rates. RVFV possesses significant pathogenic capacity in ruminants and humans, with both being susceptible to infection from the bite of RVFV-infected mosquitoes [1].

RVFV is an enveloped virus with a single-strand RNA (ss-RNA) genome with three different segments: L (large) segment, M (medium) segment, and S (small) segment. L segment encodes for the viral RNA-dependent RNA polymerase (RdRp); the M segment encodes for the glycoprotein precursor (GPC) and the nonstructural protein NSm; the S segment encodes for the nucleocapsid protein N and nonstructural protein (NS) [2]. GPC, after a post-translationally modification, forms the aminoterminal glycoprotein Gn (Gn) and the class II fusion protein Gc (Gc); these two viral proteins form a complex that is fundamental for the viral fusion to the host cell. Recently it has been demonstrated that the Gn/Gc viral complex binds to the LDL receptor-related protein 1 (Lrp1) host cell protein, which is strictly conserved through cell types and species [3]. Indeed, the RVFV cellular tropism is very broad. Previous studies demonstrated that different cell types, such as neurons, hepatocytes, and mononuclear phagocytic cells, represent the major target of RVFV in vivo, giving a high variability of symptoms after infection [1].

High tissue tropism of RVFV has also been proved in vitro; some studies demonstrated the ability of this virus to infect trophoblast cell lines (A3 and Jar) [4] and HepG2 cell lines [4,5,6,7].

Indeed, RVFV is endemic to certain African regions, but due to its high potential for spread to non-endemic areas, it is categorized as a priority pathogen and included in the World Health Organization (WHO) and Centers for Disease Control and Prevention’s (CDC) Bluepoint list [8]. In addition, RVFV is classified as a Category A Priority Pathogen by the National Institute of Allergy and Infectious Diseases in the United States of America whereby the set-up of experimental procedures to ensure safe experimental laboratory protocols is fundamental, especially in medical facilities that do not have biosafety level 3 (BSL-3) containment to handle the wild-type virus. Inactivation methods are, usually, reproducible across virus families but often they are not standardized and could be different between laboratories. To this aim, evaluating different procedures based on culturing or growth evaluation could be useful for finding a safety workflow. In fact, European Standards and specific testing protocols are only available for chemical inactivation, despite the wide use of other inactivation methods [8].

For RVFV, with regard to chemical inactivation methods, it is been established that 4% paraformaldehyde, Trizol LS, and RNA isolation kits with guanidine hydrochloride and guanidine thiocyanate as an AVL buffer are effective at inactivating RVFV in both mosquito carriers and mouse microglial cells infected by RVFV [9,10]. Although heat treatment is another simple and commonly used method for inactivating viruses, a specific protocol is currently unknown [11,12,13]. This process inactivates viruses primarily by denaturing the secondary structure of proteins critical for viral entry, thus disrupting their functional abilities [14].

In this study, we assess the ability of RVFV to infect various cell lines and examine its susceptibility to different heat inactivation protocols in order to find simple procedures that allow for viral molecular analysis on an open bench. The heat inactivation experiments were conducted using RVFV harvested from multiple cell cultures.

## 2. Materials and Methods

### 2.1. Cell Lines

HEP-2 (Human Epidermoid carcinoma; ATCC), HUH-7 (hepatocellular carcinoma; ATCC), A549 (Lung cancer; ICLC Cell Factory IST), JEG3 (Choriocarcinoma; kindly provided by Prof. Luisa Campagnolo) and VeroE6 (African green monkey kidney; ATCC) were grown in Modified Eagle Medium (MEM), 10% Fetal Bovine Serum (FBS), L-glutamine, streptomycin (100 μg/mL) and penicillin (100 U/mL) in 5% CO_2_ at 37 °C. HTR-8/SVneo (Trophoblast; kindly provided by Prof. Luisa Campagnolo) was grown in RPMI 1640, 10% Fetal Bovine Serum (FBS), L-glutamine, streptomycin (100 μg/mL) and penicillin (100 U/mL) in 5% CO_2_ at 37 °C.

### 2.2. Viral Production

RVFV was obtained from the National Collection of Pathogenic Viruses (NCPV) and propagated in VeroE6, HEP-2, HUH-7, A549, JEG3 and HTR-8/SVneo cell lines. For virus production, confluent cell monolayers were washed twice with PBS 1X and infected with RVFV at a multiplicity of infection (m.o.i.) of 0.5 Tissue Culture Infective Dose (TCID)_50_/cell. After the adsorption period (1 h), the viral inoculum was removed, and cells were cultured for 48 h. At 48 h post-infection (h.p.i.), the viral suspension was collected, after 3 freezing/thawing cycles, clarified by centrifugation at 2000 rpm for 10 min, aliquoted, and stored at −80 °C. Virus titers were determined by limiting dilution assay, and residual infectivity was expressed as 50% TCID_50_/mL, calculated according to the Reed and Muench method. Viral infection and production were performed in a BSL-3.

### 2.3. Heat Inactivation

To inactivate the virus, 400 µL aliquots of viral stocks from various cell lines were heated at 60 °C, 70 °C, 80 °C, and 95 °C at different time points using a Labnet thermoblock machine. The time points used for 60 °C were 1, 5, 10, 15, 30, 45 and 60 min. The time points used for 70 °C, 80 °C and 95 °C were 1, 5, 10, 15 min. The temperature was controlled using a thermometer. After heating, the samples were chilled on ice for 3 min. After inactivation, the samples were analyzed by TCID_50_ assay and RT-PCR. Each inactivation condition was run and analyzed at least in triplicate. To determine the fastest inactivation condition, viral stocks were also exposed to 95 °C for 1, 2 and 3 min. In other cases, the viral stock was diluted with 1X PBS and subjected to heat treatment at 95 °C for 5 min, as previously described. Subsequently, these samples were examined using RT-PCR.

To evaluate the heat-inactivation method in human sample, urine samples were spiked with RVFV at different concentrations (10^5.5^, 10^4.5^, 10^3.5^, 10^2.5^, 10^1.5^ viral RNA copies/mL) and then heated at 60 °C for 1 h, 70 °C for 15 min, 80 °C for 10 min and 95 °C for 5 min. Subsequently, these samples were examined using RT-PCR. The inactivation was then confirmed using the spiked sample on VeroE6 cells.

### 2.4. Median TCID_50_ Assay

Vero E6 cells were seeded in 96-well tissue culture plates at a density of 10 × 10^3^ cells/well in a growth medium containing 10% FCS and incubated at 37 °C in a humidified atmosphere with 5% CO_2_. After 24 h, semi-confluent cell monolayers were infected with serial dilutions of viral suspension, in a growth medium containing 2% FCS (four replicates for each dilution) and incubated at 37 °C. At 6 days after infection, the virus-induced cytopathic effect was evaluated. TCID_50_ infectivity assay was performed in a BSL-3.

### 2.5. Viral Quantification by RT-PCR

Nucleic acids were extracted from the supernatant of infected cells using QIAamp^®^ Viral RNA (QIAGEN, Hilden, Germany) according to manufacturing instructions. Briefly, 140 µL of supernatant that contains viral particles were added to 560 µL of buffer AVL-containing carrier. After washing with AW1 and AW2 buffer, nucleic acids were eluted with 60 µL of AVE buffer. RVFV RNA was detected by the commercial assay RealStar^®^ Rift Valley Fever Virus RT-PCR Kit 1.0 (Altona, Hamburg, Germany) [15].

Assay conditions were as follows: reverse-transcription 55 °C for 20 min, denaturation 95 °C for 2 min, then 45 cycles of 95 °C for 15 s, 55 °C for 45 s, 72 °C for 15 s [16]. It has already been demonstrated that this RT-PCR method did not cross-react with Dengue Virus, JEV, St. Louis Encephalitis Virus, Usutu Virus, Marburg Virus, Ebola Virus, West Nile Virus, Yellow Fever Virus, nor Zika Virus [17]. The analytical assay sensitivity was 890 copies/mL. The precision data were determined by evaluating an intra-assay, inter-assay and inter-lot variability. The coefficient of variation of the test is 1.10%, as indicated in the Altona brochure.

An RVFV standard RNA, which was provided by Altona Diagnostic, was serially diluted to a theoretical range of 10^6^ to 10^−1^ copies in order to generate a standard curve. The interpolation of the data was performed using GraphPad Prism version 9 (GraphPad Software, La Jolla, CA, USA) using Sigmoidal, 4PL model.

### 2.6. Measure of Inactivation Efficiency

The effectiveness of thermal treatment at various temperatures in decreasing viral infectivity or activity was assessed using inactivation efficiency, which is defined as the proportion of viruses rendered inactive relative to the time and temperature of exposure.

The formula used for evaluating the inactivation efficiency of the thermal treatment was:

$$Inactivation\;efficiency\;(\%)=\left(1-\frac{V_{t}}{V_{0}}\right)\cdot 100$$

where V_t_ represents the viral titer measured at time point (t) post-treatment, and V_0_ is the initial viral titer before treatment.

### 2.7. Determination of D- and Z-Values

The thermal inactivation kinetics of the RVFV across different cell lines were characterized by calculating the decimal reduction time (*D*-value) and the thermal resistance constant (Z-value).

The *D*-value was defined as the time required at a specific temperature to achieve a one-logarithm reduction in viral titer. This was determined by plotting the logarithm (base 10) of the surviving viral titer against time for each test temperature. The *D*-value was then calculated as the negative inverse of the slope of the resulting plot, with the line of best fit for the survivor curves established through regression analysis.

To calculate the Z-value, which indicates the temperature change needed to effect a tenfold change in the *D*-value, the linear regression of the logarithm (base 10, Log) of the *D*-values was performed against their corresponding heating temperatures. The absolute values of the inverse of the slope were then used to calculate the Z-values.

### 2.8. Statistical Analyses

Statistical analyses and graphical representations were performed using GraphPad Prism version 9 (GraphPad Software, La Jolla, CA, USA). Differences among the virus derived from the cell lines were assessed using one-way repeated measures ANOVA. Tukey’s post-hoc test was applied for multiple comparisons where appropriate, and unpaired Student’s *t*-test was used for specific pairwise comparisons. Differences were considered significant if the adjusted *p*-value was less than 0.05.

## 3. Results

### 3.1. Infection of Various Cell Lines with RVFV

**Figure 1:**
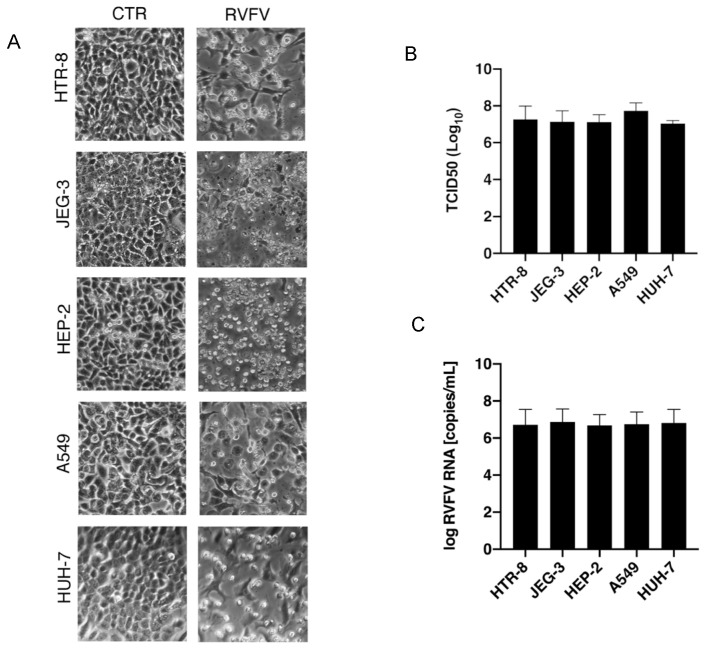
In vitro replication of RVFV and its cytopathic effect. The cell lines HTR-8, JEG-3, HEP-2, A549, and HUH-7 were exposed to RVFV with a multiplicity of infection (m.o.i) of 0.5 for a period of 48 h. The resulting infection displayed (**A**) pronounced cytopathic effects (CPE). CTRL = uninfected sample, RVFV = infected sample. The images represent one of three similar experiments. Viral replication at 48 h post-infection was verified in the infected cells using (**B**) TCID_50_ assay and (**C**) quantitative RT-PCR. The histograms represent, respectively, the quantification of the viral infectious particles and the copies/mL of the viral RNA. Data are represented as the mean relative to the control plus S.D.

To evaluate the tropism of RVFV in vitro, we infected five cell lines (HTR-8, JEG-3, HEP-2, A549, and HUH-7) with RVFV at m.o.i. of 0.5. The analyses of the produced viral particles demonstrated that RVFV is detected in the cellular supernatants starting from 8 h post-infection (h.p.i.), causing a strong cytopathic effect (CPE) only after 48 h.p.i. (Figure 1A), an experimental point in which RVFV reached its peak titer in all the cell lines analyzed. The quantification of the viral infectious particles, measured by TCID_50_ assay at 48 h.p.i., confirmed the capability of the virus to similarly replicate in all the cell lines analyzed, with a high yield (mean titer at 48 h.p.i. = 10^7.26^ copies/mL) (Figure 1B). The evaluation of viral particles released was also confirmed by RT-PCR (mean RNA copies/mL at 48 h.p.i.: 10^6.76^ copies/mL) (Figure 1C).

### 3.2. Efficacy of Heat Treatment on RVFV Inactivation

**Figure 2:**
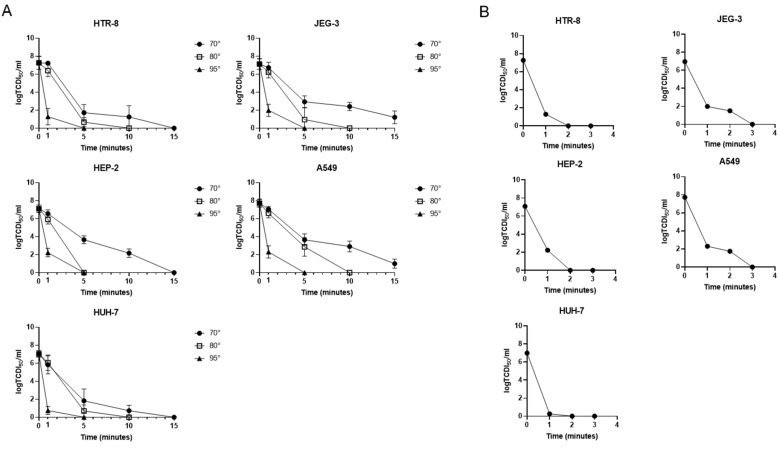
RVFV infectivity after heat treatment. To evaluate heat treatment on RVFV inactivation, the stocks derived from the different cell lines (HTR-8, JEG-3, HEP-2, A549 and HUH-7) were incubated at 70 °C, 80 °C, and 95 °C for 1, 5, 10, and 15 min. (**A**) The viral infectious particles were evaluated by TCID_50_ assay in all the conditions tested. To find the quickest condition of inactivation, (**B**) the viral infectious particles, measured by TCID_50_ assay, were also valued in samples treated at 95 °C for 1, 2, and 3 min. The histograms represent, respectively, the quantification of the viral infectious particles. Data are represented as the mean relative to the control plus S.D.

Thermal treatment is widely used for viral inactivation; nevertheless, its efficacy is highly variable for different RNA viruses, even within the same family [18,19]. To determine the thermal stability of RVFV obtained from the different cell lines, we evaluated the efficacy of different conditions: 70 °C, 80 °C, and 95 °C for 1, 5, 10, and 15 min. Additionally, as recommended for serological analyses, we also analyzed a 60 °C heat treatment for longer time points. In the latter, the results demonstrated that RVFV was stable also after 60 min of heat treatment at 60 °C with infectivity stable at 2 Log (Figure S1). Instead, the analyses of the inactivation kinetics at 70 °C highlighted a gradual decrease in the viral infectious particles obtained from JEG-3, HEP-2, and A549 cell lines (Figure 2A). Conversely, regarding the virus obtained from HTR-8 and HUH-7 cells, a sharp decrease in the viral infectivity was observed already after 5 min of treatment (inactivation efficiency of 76.2% and 74%, respectively) (Table 1), even though no complete abolishment was observed at this time point. The differences between the inactivation kinetics at 70 °C of the virus derived from the different cell lines were confirmed by statistical analyses (*p* = 0.004); the higher statistical significance was measured between HTR-8 vs. A549 (*p* = 0.048), JEG-3 vs. HUH-7 (*p* = 0.041) and A549 vs. HUH-7 (*p* = 0.037). The 80 °C and 95 °C inactivation, on the contrary, did not underline significant differences between the stocks derived from the different cell lines (*p* = 0.19 and *p* = 0.28, respectively).

These findings are supported by the *D*- and *Z*-values calculated at 70, 80, and 95 °C for all cell lines (Table S1). Specifically, at 70 °C, the *D*-values for JEG-3 and A549 were 2.52 and 2.30 min, respectively, which were higher than those of the other cell lines, while HTR8 showed the lowest *D*-value at 1.99 min. At 80 °C, HTR8, JEG-3, A549, and HUH-7 demonstrated comparable *D*-values of 1.30, 1.34, 1.29, and 1.36 min, respectively. In contrast, HEP-2 showed a significantly lower *D*-value of 0.69 min, indicating reduced stability at this temperature. The differences in *D*-values at 95 °C were less pronounced; HTR8, HEP-2, and HUH-7 shared identical *D*-values of 0.28 min, while JEG-3 and A549 had *D*-values of 0.46 and 0.42 min, respectively.

Moreover, the *Z*-values for HTR8 (28.4 °C), HEP-2 (29.0 °C), and HUH-7 (27.7 °C) were lower than those for JEG-3 (33.6 °C) and A549 (33.7 °C), suggesting that these cell lines exhibit greater sensitivity to thermal changes.

In addition, heat treatment at 70 °C and 80 °C required at least 15 and 10 min, respectively, to cause the maximum efficacy of virus inactivation, instead treatment at 95 °C was able to abolish the viral titer already after 5 min. For this reason, we also analyzed the efficacy of inactivation at 95 °C considering intermediate time points (1, 2, and 3 min), demonstrating that RVFV lost its infection ability already after 3 min of treatment, suggesting that this could be an optimal experimental condition for heat inactivation. The abolishment of the infectious ability was further confirmed using the 95 °C heat-inactivated viral stocks onto the same cells used for the production. As shown in Supplementary Figure S2, no cell lines showed any cytopathic effect (Figure S2).

### 3.3. RVFV RNA Detection After Heat Inactivation

**Figure 3:**
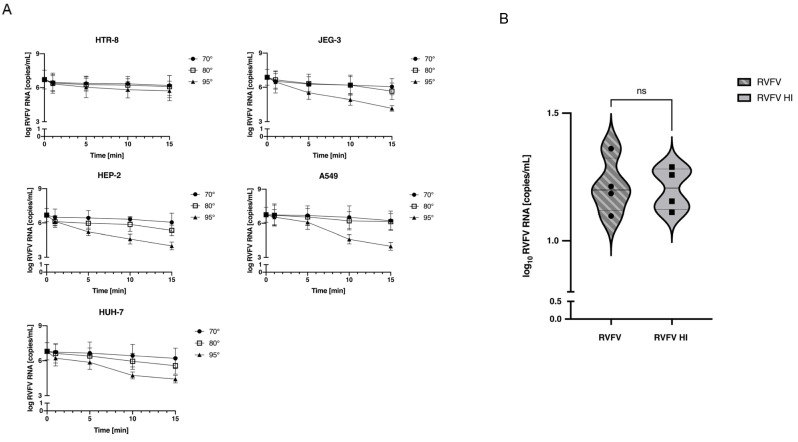
RVFV RNA detection after heat inactivation. To estimate the denaturation of viral RNA after heat treatment at different conditions RVFV RNA was measured by RT-PCR (measured as RNA copies/mL) in (**A**) stocks derived from the different cell lines incubated at 70 °C, 80 °C and 95 °C for 1, 5, 10, 15 min and (**B**) in diluted stocks to evaluate denaturation in the presence of few copies of viral RNA (mean RNA copies/mL: 10^1.2^ copies/mL). The histograms represent the expression of viral RNA measured as copies/mL. Data are represented as the mean relative to the control plus S.D. ns = not significative.

Elevated temperatures can, under specific circumstances, denature a virus’s genetic material, resulting in undetectable viral RNA [20]. To check if the RNA remained intact after heat treatment, we quantified it in all tested conditions via titration with RT-PCR. Figure 3A demonstrates that the viral RNA was detectable across all tested conditions, exhibiting greater denaturation exclusively in the final two conditions (average of RNA copies/mL of 1.83 Log and 2.31 Log at 95 °C for durations of 10 and 15 min, respectively). Importantly, the optimal experimental condition discovered through TCID_50_ assay (being exposed to 95 °C for 5 min) resulted in minor degradation of RVFV RNA, with an average difference of copies/mL post- and pre-heat treatment of 1 Log. This suggests that such a thermal condition is suitable for inactivating samples during pre-analytic processing. To determine whether this thermal protocol is also effective for samples containing low levels of viral RNA, we subjected a diluted viral stock to RT-PCR analysis before and after applying the heat treatment. As demonstrated in Figure 3B, detection of the RVFV genomic RNA was still possible, confirming the potential of this method for ensuring the safe handling of clinical and research specimens.

### 3.4. RVFV Inactivation in Human Samples

**Figure 4:**
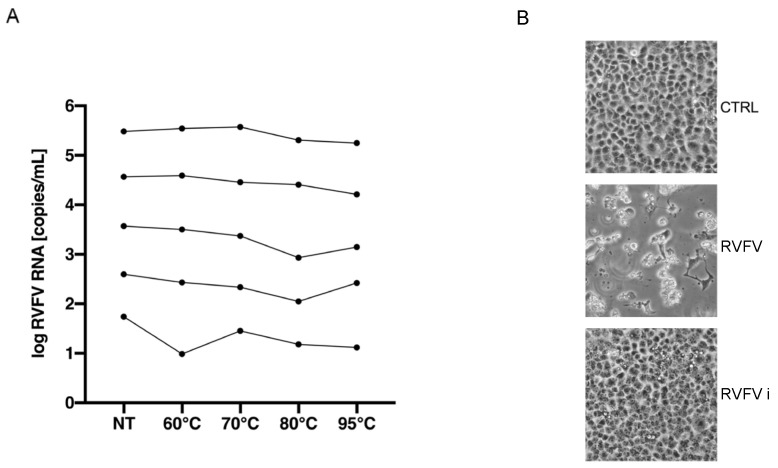
RVFV heat inactivation in human samples. To evaluate the reliability of the method in human samples, urine samples were spiked with RVFV at different concentrations (respectively, 10^5.5^, 10^4.5^, 10^3.5^, 10^2.5^, 10^1.5^ viral RNA copies/mL) and then heated at 60 °C for 1 h, 70 °C for 15 min, 80 °C for 10 min and 95 °C for 5 min. (**A**) RVFV RNA stability was determined by RT-PCR (measured as RNA copies/mL) NT = Not Treated Sample. (**B**) The images show the abolishment of the infectious ability of 95 °C inactivated samples on Vero E6 cell lines. CTRL = uninfected sample, RVFV = infected with spiked-sample, RVFVi = infected with 95 °C inactivated spiked-sample. The images are representative of multiple experiments.

To evaluate the heat-inactivation method in a human sample, urine samples were spiked with RVFV at different concentrations (respectively, 10^5.5^, 10^4.5^, 10^3.5^, 10^2.5^, 10^1.5^ RVFV RNA copies/mL) and then heated at 60 °C for 1 h, 70 °C for 15 min, 80 °C for 10 min and 95 °C for 5 min. We, firstly, evaluated the degradation of viral RNA using various starting concentrations. As shown in Figure 4A, also in human samples, the RVFV RNA remained detectable in all conditions tested and showed high stability under all heating conditions. The abolishment of the infectious ability was further confirmed using the 95 °C heat-inactivated spiked-samples on Vero E6 (Figure 4B).

## 4. Discussion

This study was undertaken to confirm the high infectivity of RVFV in different cell lines and to define a heat-inactivation protocol that can be easily used to maintain a safe handling procedure. Analyzing viruses’ tropisms is crucial to understanding disease manifestations and finding important insights into the prevention and treatment of viral disease, particularly for viruses considered a priority pathogen worldwide. As already demonstrated in other works [4,7,21], our data confirmed that RVFV can replicate in the human hepatic cell line (HUH-7) and trophoblast cell line (HTR-8). Additionally, we also demonstrated the ability of RVFV to replicate, with a high yield, in human epidermoid (HEP-2), lung (A549), and placenta (JEG-3) cell lines (Figure 1A), suggesting that the elevated tissue tropism of RVFV can strongly influence its potential for spreading.

In this paper, we also set up a heat inactivation protocol to increase safe laboratory practice for medical and research samples. It has been well-documented that chemical inactivation is an effective method for neutralizing RVFV [9,10]. However, this process involves the manipulation of potentially live RVFV samples, which poses a risk to handlers. Typically, during the pre-analytical phase, viral inactivation is carried out using both chemical and heat treatments concurrently. Here, we assessed the thermal resistance and infective capacity of RVFV particles, produced in various cell cultures, when exposed to temperatures of 60 °C, 70 °C, 80 °C, and 95 °C. Our analyses demonstrated a high stability of viral particles at 60 °C with a maintained infectivity also after 60 min of treatment (Figure S1). The data suggest the necessity of using higher temperatures to obtain a safe workflow. The data collected at 70 °C show a greater time-dependent decline in the virulence of infectious particles from JEG-3, HEP-2, and A549 cell lines. Specifically, after 10 min, the inactivation rates were 66.2% for JEG-3, 69.5% for HEP-2, and 62.4% for A549 cells. Despite these trends, there was variability in the inactivation outcomes after 15 min, with incomplete inactivation observed in viral particles from JEG-3 and A549, while those from HEP-2 were entirely inactivated. Otherwise, at 70 °C, the viral particles produced in HTR-8 and HUH-7 showed a higher inactivation efficiency (76.2% and 74%, respectively, already after 5 min of treatment) with a complete inactivation after 15 min of treatments (Figure 2A and Table 1), suggesting this as a possible inactivation condition for serological analyses. This phenomenon can be explained by the presence of increased binding of viral particles to cell debris, which may hinder or delay the inactivation of viral particles [22]. Contrastingly, the inactivation rates for viral stocks cultured from various cells showed no substantial differences at 80 °C and 95 °C, with roughly 89% inactivation at 80 °C following 5 min of treatment, and total loss of viral infectivity at 95 °C after just 3 min (Figure 2A,B, Figure S2 and Table 1). The data obtained are in line with the protocol defined for other RNA viruses such as SARS-CoV-2 in which it has been demonstrated that the fastest heat condition to obtain the loss of infectivity is 95 °C for 3 min [11].

The effectiveness of heat treatment is commonly quantified using *D*- and *Z*-values, which represent the time required to reduce the viral population by one log (90%) at a specific temperature and the temperature change necessary to achieve a tenfold change in the *D*-value, respectively. The calculated *D*- and *Z*-values provide a quantitative framework for assessing the thermal sensitivity of the various cell lines studied.

At 70 °C, the *D*-values indicated that JEG-3 and A549 exhibited greater thermal resistance compared to the other cell lines, while HTR8 demonstrated higher sensitivity (*D*_70_ = 1.99 min). At 80 °C, all cell lines showed increased sensitivity, as evidenced by lower *D*-values; notably, HEP-2 displayed reduced thermal resistance (*D*_80_ = 0.69 min). Finally, at 95 °C, the differences among cell lines become less distinct. The *Z*-values further confirm the heightened thermal sensitivity of HTR8, HEP-2, and HUH-7 relative to JEG-3 and A549, which require larger temperature changes to achieve the same inactivation effect. A critical aspect of this study is the lack of comparable data in the literature, making it challenging to place these results in a broader context (Table S1).

Heat, known for causing changes in viral receptors, can also influence the denaturation of viral RNA [23]. Our research also showed the effect of thermal inactivation on the integrity of the RVFV genome under the different conditions tested (i.e., with a high or low number of viral RNA copies) (Figure 3A,B). The results of heating on the viral genome have been already described for other RNA viruses such as SARS-CoV-2 and Nipah Virus [19,24,25]. In our study, the greatest level of denaturation was observed following the last two conditions (95 °C for 10 and 15 min). In these instances, the differences in the RVFV RNA copies/mL before and after heat treatment were 1.83 Log and 2.31 Log, respectively. On the other hand, a 95 °C heat treatment for 5 min resulted in only minor denaturation of the viral RNA, with an average difference in RNA viral copies/mL of 1 Log between untreated and heat-treated viruses. In addition, data obtained on human samples also demonstrated the reliability of the 95 °C heating treatment on the infectivity of RVFV. Although RVFV RNA is highly stable at different heating conditions, 95 °C heat treatment causes the complete loss of the infectious ability (Figure 4A,B). This indicates that brief heat treatment could be an efficient protocol for inactivating RVFV in clinical and research samples used for molecular analyses, especially in healthcare settings lacking high-level containment laboratories.

## 5. Conclusions

In conclusion, the data presented in this study could represent a heat protocol to inactivate not only RVFV but also samples that should be tested for other viral hemorrhagic fever infections. The ideal solution could be to inactivate samples with heat, as the first step in the process, to render the workflow safer and to perform the subsequent molecular analysis protocol on an open bench.

## Figures and Tables

**Table 1:** Inactivation efficiency (IE) values for the cell lines at different temperatures during thermal treatment.

Temperature [Celsius]	Time [min]	HTR8	IE (%)	JEG-3	IE (%)	HEP-2	IE (%)	A549	IE (%)	HUH-7	IE (%)
70 °	0	7.27		7.14		7.12		7.73		7.04	
	1	7.23	0.6%	6.73	5.7%	6.56	7.9%	7.05	8.8%	5.83	17.2%
	5	1.72	76.2%	2.94	58.8%	3.66	48.6%	3.65	52.8%	1.83	74.0%
	10	1.25	82.8%	2.41	66.2%	2.17	69.5%	2.91	62.4%	0.73	89.6%
	15	0	100.0%	1.21	83.1%	0	100.0%	1.00	87.1%	0	100.0%
80 °	0	7.27		7.14		7.12		7.73		7.04	
	1	6.40	12.0%	6.21	13.0%	5.93	16.7%	6.61	14.5%	6.06	13.9%
	5	0.67	90.8%	0.95	86.7%	0	100.0%	2.87	62.9%	0.70	90.1%
	10	0	100.0%	0	100.0%			0	100.0%	0	100.0%
	15										
95 °	0	7.27		7.14		7.12		7.73		7.04	
	1	1.29	82.3%	1.99	72.1%	2.23	68.7%	2.30	70.2%	0.75	89.3%
	5	0	100.0%	0	100.0%	0	100.0%	0	100.0%	0	100.0%
	10										
	15										

## Data Availability

The original contributions presented in this study are included in the article and Supplementary Materials. Further inquiries can be directed to the corresponding author.

## Supplementary Materials

The following supporting information can be downloaded at: https://www.mdpi.com/article/10.3390/pathogens13121089/s1, Figure S1—RVFV infectivity analyses after 60 °C thermal treatment. The viral infectious particles were evaluated by TCID_50_ assay after 60 °C thermal treatment at different time points. The histograms represent respectively the quantification of the viral infectious particles. Data are represented as the mean relative to the control plus S.D. Figure S2—RVFV infectivity analyses after 95 °C thermal treatment by evaluating the cytopathic effect. The abolishment of infection ability of the samples treated at 95 °C was confirmed using the inactivated viral stocks onto the same cell lines used for the production. CTRL = uninfected sample, RVFVi = 95 °C inactivated infected sample. The images are representative of multiple experiments. Table S1—Decimal reduction times (D-values) and Z-values calculated at 70, 80, and 95 °C for all cell lines. R^2^ represents the correlation coefficient.
