# Supplementary material for: Heat Treatment as a Safe-Handling Procedure for Rift Valley Fever Virus
Source: Pathogens. 2024 Dec 10;13(12):1089. doi: 10.3390/pathogens13121089 (PMC11676096; doi:10.3390/pathogens13121089)
Supplement: Supplementary file 1 [file pathogens-13-01089-s001.zip › Supplementary/Supplementary Table1.pdf]

Table S1. Decimal reduction times (D-values) and Z-values calculated at 70, 80, and 95 °C for all cell lines. R2 represents the correlation coefficient.

	Heat treatment temperature [°C]							
	70°C		80°C		95°C			
Cell line	D-value [min]	R^2	D-value [min]	R^2	D-value [min]	R^2	Z-value [°C]	R^2
HTR8	1.99	0.8288	1.30	0.8531	0.28	0.8782	28.4	0.9587
JEG-3	2.52	0.8701	1.34	0.877	0.46	0.8284	33.6	0.9988
HEP-2	2.13	0.9763	0.69	0.9989	0.28	0.9555	29.0	0.9689
A549	2.30	0.9274	1.29	0.9825	0.42	0.8436	33.7	0.9952
HUH-7	2.16	0.8518	1.36	0.8578	0.28	0.8289	27.7	0.9651
