# Supplementary figures and images for: Heat Treatment as a Safe-Handling Procedure for Rift Valley Fever Virus
Source: Pathogens. 2024 Dec 10;13(12):1089. doi: 10.3390/pathogens13121089 (PMC11676096; doi:10.3390/pathogens13121089)

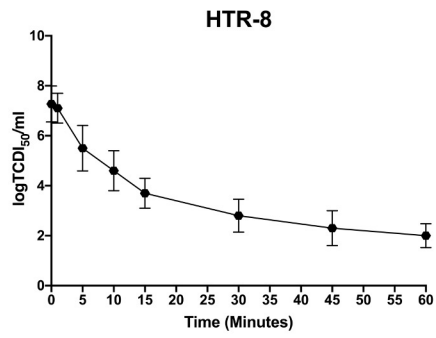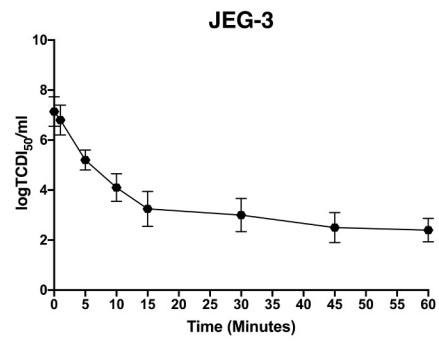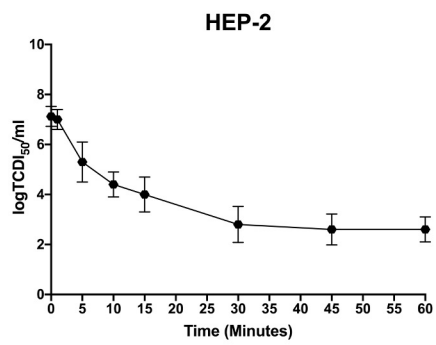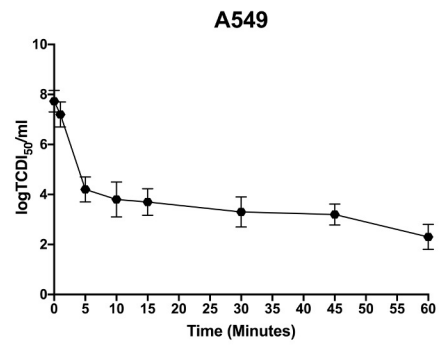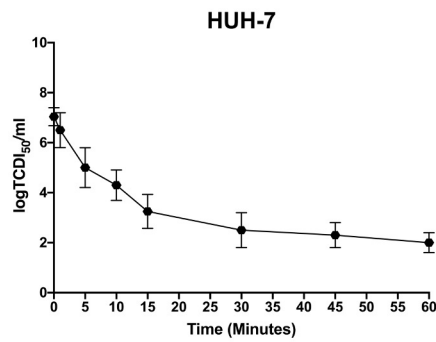

Figure S1

Supplement: Supplementary file 1 [file pathogens-13-01089-s001.zip › Supplementary/Figure S1.pdf]

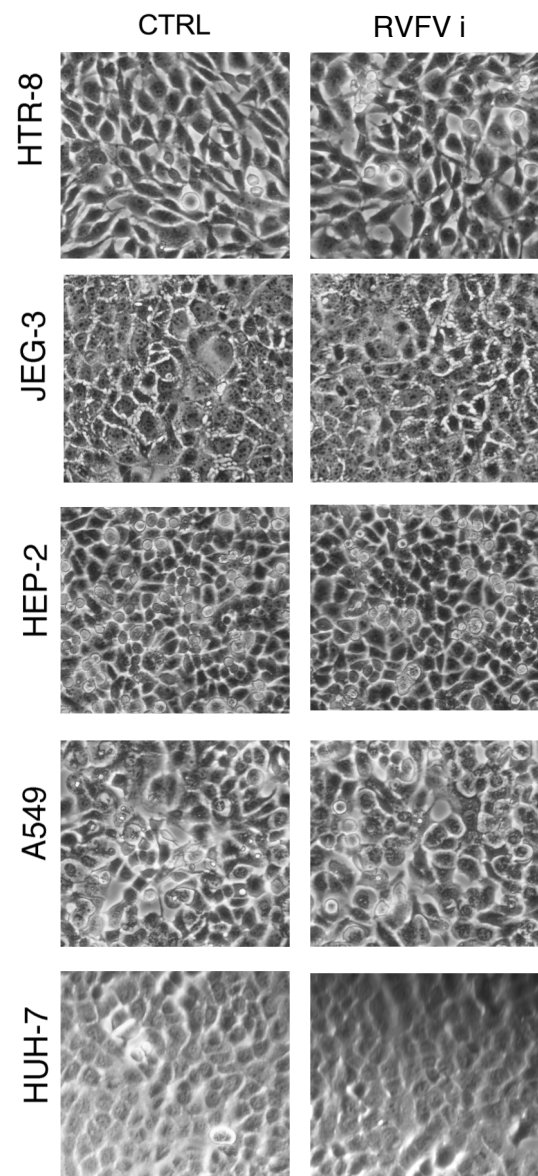

Figure S2

Supplement: Supplementary file 1 [file pathogens-13-01089-s001.zip › Supplementary/Figure S2.pdf]
